# High viral loads of human papillomavirus predict risk of invasive cervical carcinoma

**DOI:** 10.1038/sj.bjc.6602436

**Published:** 2005-03-01

**Authors:** M Moberg, I Gustavsson, E Wilander, U Gyllensten

**Affiliations:** 1 Department of Genetics and Pathology, Rudbeck Laboratory, Uppsala University, Uppsala 751 85, Sweden

**Keywords:** human papillomavirus, invasive cervical cancer, viral load

## Abstract

High loads of human papillomavirus (HPV) 16 and HPV 18/45 increase the risk of developing invasive cervical carcinoma, revealing higher risk in percentiles of highest viral loads for HPV 16 (odds ratio (OR) 58.7, 95% confidence interval (CI) 21.9–151.4) compared to HPV 18/45 (OR 3.3, 95% CI 1.5–7.2). Thus, HPV load is a type-dependent risk marker for invasive carcinoma.

Human papillomavirus (HPV) is a necessary cause of cervical cancer, but the high prevalence of transient infections makes detection of the presence or absence of virus an inefficient means of identifying women at risk of developing cervical cancer (Ho *et al*, 1998; Jacobs *et al*, 2000). Viral load has been suggested as a marker of nontransient infection, and high HPV load in smears with normal cytology has been associated with the risk of developing dysplasia and carcinoma *in situ* (CIS) (Josefsson *et al*, 2000; van Duin *et al*, 2002; Dalstein *et al*, 2003; Moberg *et al*, 2004). However, it is not known whether high viral load is also predictive of invasive cervical cancer, as past studies have pooled the outcomes of invasive cancers together with earlier stages of the disease (Lorincz *et al*, 2002; Sherman *et al*, 2002, 2003; Sun *et al*, 2002; Dalstein *et al*, 2003; Gravitt *et al*, 2003). Integration of high-risk HPV into the cell genome has been suggested to play a pivotal role in malignant transformation (Boshart *et al*, 1984; Cullen *et al*, 1991; Daniel *et al*, 1997). As a consequence, the association between viral load and development of CIS may not automatically imply a strong association of high viral load and progression into invasive carcinoma. Here, we determine whether viral load in cervical smears is a risk marker of later developing invasive cervical cancer.

## MATERIALS AND METHODS

Cases diagnosed with invasive squamous-cell carcinoma of the cervix and with registered smears taken at least 1 year prior to the diagnosis date were identified from the organised screening program (PAP-smear register) and the tumour register at the Clinic of Pathology, Uppsala Academic Hospital and Uppsala University, Sweden. Controls were selected from the same organised screening programme and have been described in detail elsewhere (Ylitalo *et al*, 2000). Birth years of cases and controls ranged from 1922 to 1968. Two strategies were used to select smears from both cases and controls. The screening history of individual women was examined to identify suitable smears for inclusion. First, we attempted to study an early phase of the infection. Therefore, we started from the date of diagnosis and stepped backwards in the screening history until reaching a smear fulfilling the criteria of (a) being negative itself, (b) being preceded by a normal smear or being preceded by an abnormal smear taken at least 3 years earlier, or being the first registered smear. The time span of 3 years was chosen to increase the likelihood of spontaneous regression of the preceding lesion (Schlecht *et al*, 2003), and consequently decreasing the risk of selecting false-negative smears. Secondly, up to three smears per woman were chosen from cases and controls at random to increase the chance of including an existing HPV infection. Smears collected less than 1 year prior to diagnosis were excluded. DNA was extracted from archival PAP smears using previously described methods (Josefsson *et al*, 1999), and amounts of human genomic DNA, HPV 16, HPV 31 and a combined load for HPV 18 and 45 were estimated using published methods (Moberg *et al*, 2003). Viral load is reported as HPV genomes per cell equivalent. An estimate of 6.6 pg per human cell was used to convert human DNA estimates into cell equivalents (CE). The strategy of a combined load of HPV 18 and 45 was employed to minimise the number of parallel quantitative PCR reactions. Odds ratios (ORs) were estimated by logistic regression (LOGISTIC procedure, SAS v6.12, SAS Institute Inc., Cary NC, USA).

## RESULTS

A single, normal smear was obtained from 62 cases and 501 controls (Table 1). The smears were collected on average 9 years (range 1–28) prior to the diagnosis date and classified as negative for squamous cell abnormalities. A total of 10 cases had registered abnormal cytology during the screening interval before the collection of the normal smear included in this study. Four cases had diagnoses equivalent to ASCUS or LSIL, and six cases HSIL, as the worst registered lesion prior to the normal smear. However, in these four cases, the smear taken closest before the selected smear was classified as negative for lesions.

In all, 45% of cases and 6% of controls test positive for HPV 16 (Table 1). In estimating the risk, crude ORs are presented since initial introduction of birth year and age at smear collection in the regression model did not alter the estimates appreciably. Odds ratio increases with HPV 16 viral load, reaching a maximum of OR=51 in the percentile with the highest viral load (Table 2). HPV 31 is found in 8% of cases and 4% of controls and HPV 18/45, detected together, are found in 13% of cases and 6% of controls. The ORs for HPV 31 and HPV 18/45 are significant for the percentiles with the highest viral load (Table 2).

The random set includes 139 cases (261 smears) and 548 controls (1049 smears) (Table 1). In all, 168 (64%) of the included smears from cases were classified as negative for malignant cells. The corresponding number for controls is 819 (78%). Cytological information was missing in the archives for a total of five smears from cases and 188 smears from controls. In total, 63% of cases and 10% of controls test positive for HPV 16 (Table 2). The OR, calculated from the mean HPV 16 viral load per woman, increased with viral load and reached a maximum of OR=59 for the percentile with the highest viral load (Table 2). The ORs for HPV 31 are not significant, while the OR for HPV 18/45 is significant for the percentile with the highest viral load (Table 2).

## DISCUSSION

**Figure 1:**
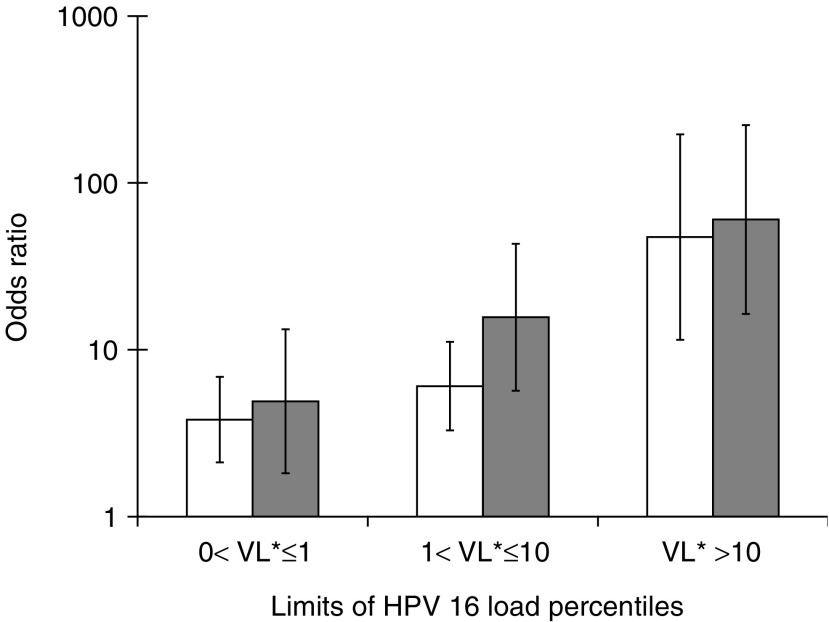
Odds ratio with 95% CI of cervical CIS (empty bars) or invasive cervical carcinoma (filled bars) depending on HPV 16 load. VL^*^ represents the number of HPV 16 genomes per human cell equivalent.

Our results indicate that increasing viral load of HPV 16 in cervical smears increases the risk of future invasive cervical cancer. For HPV 18/45, a significant OR was only seen for the highest viral-load percentile. The relationship between OR of invasive cancer and viral load of HPV 16 is similar to that previously described for CIS (Moberg *et al*, 2004) (Figure 1); consistent with this, CIS and invasive cervical cancer reflect the same basic aetiology. Thus, our results show that a high viral load in PAP smears is a risk factor not only for the development of CIS but also for invasive cancer. Our data support the hypothesis of Peitsaro *et al* (2002) that high viral loads are likely to increase the risk for events initiating dysplasia such as viral integration. Given that integration is a random event, the risk for such an event would increase with high viral loads, independent of whether a high viral load reflects widespread infection or high copy numbers per cell. HPV 16 displays a generally higher risk for invasive cervical cancer development over viral load categories than HPV 18 and HPV 31. Interestingly, HPV 16 appears to be able to induce malignant transformation without integration (Pirami *et al*, 1997; Badaracco *et al*, 2002; Hudelist *et al*, 2004). This indicates that additional factors to integration may be important for malignant transformation. Longitudinal studies including both the physical state and load of the virus are needed in order to determine their relative importance for development of cervical cancer. Judging from our data, HPV load provides information about the risk for subsequent development of invasive carcinomas, but the extent correlates strongly with the HPV type.

## Figures and Tables

**Table 1:** Characteristics of the case-control material

	**Single smear cohort**		**Random smear cohort**	
	**Cases**	**Controls**	**Cases**	**Controls**
***n***	**62**	**501**	**139**	**548**
*Year of birth*				
Mean	1946.8	1949.0	1946.6	1949.0
Range	1922–1965	1922–1968	1922–1968	1922–1968
				
*Age at smear collection*				
Mean	34.7	30.9	34.6	29.8
Range	20–62	15–63	15–62	15–63
				
*Human DNA*				
Mean^a^	159.8	219.2	175.7	206.2
Range	5.0–1354	5.4–6264	5.0–2465	5.0–15914
				
*HPV 16*				
No of positive women	28	28	87	56
No of positive smears	28	28	120	66
Mean viral load^b^	138.3	17.7	97.0	12.9
Range	0.05–2812	0.02–300.3	0.03–2812	0.01–300.3
				
*HPV 31*				
No of positive women	5	19	9	34
No of positive smears	5	19	15	39
Mean viral load^b^	17.7	37.4	29.8	50.5
Range	0.50–36.7	0.03–543.1	0.3–147.2	0.02–762.6
				
*HPV 18/45*				
No of positive women	8	28	28	55
No of positive smears	8	28	39	64
Mean viral load^b^	157.6	77.2	265.3	71.4
Range	0.11–1158	0.01–1402	0.02–5105	0.01–2044

a Mean estimated number of human cells equivalents per reaction.

b Mean number of HPV genomes per human cell equivalent.

**Table 2:** Odds ratios for invasive cervical carcinoma in relation to the mean normalised viral load of individual HPV types or group of closely related HPV types

			**95% CI**		
**Limits**	**Cases/controls**	**OR**	**Lower limit**	**Upper limit**	***P*-value**
Single smear cohort					
HPV 16	34/473	Ref.^b^	—	—	—
Negative	4/10	5.565	1.658	18.673	0.0055
0<VL^a^⩽0.44	5/9	7.729	2.454	24.342	0.0005
0.44<VL^a^⩽2.47	8/6	18.549	6.087	56.524	0.0001
2.47<VL^a^⩽18.22	11/3	51.010	13.583	191.560	0.0001
18.22<VL^a^					
					
HPV 31	57/482	Ref.^b^	—	—	—
Negative	0/7	0	0	999	0.9866
0<VL^a^⩽0.50	2/7	2.416	0.490	11.910	0.2785
0.50<VL^a^⩽10.25	3/5	5.074	1.181	21.791	0.0290
10.25<VL^a^					
					
HPV 18/45	54/473	Ref.^b^	—	—	—
Negative	2/10	1.752	0.374	8.205	0.4767
0<VL^a^⩽0.11	2/9	1.947	0.410	9.243	0.4021
0.11<VL^a^⩽3.40	4/9	3.893	1.160	13.068	0.0278
3.40<VL^a^					
					
Random smear cohort					
HPV 16	52/492	Ref.^b^	—	—	—
Negative	13/23	5.348	2.557	11.183	0.0001
0<VL^c^⩽0.49	18/18	9.462	4.637	19.304	0.0001
0.49<VL^c^⩽3.09	25/10	23.654	10.767	51.967	0.0001
3.09<VL^c^⩽21.96	31/5	58.662	21.866	151.377	0.0001
21.96<VL^c^					
					
HPV 31	130/514	Ref.^b^	—	—	—
Negative	2/13	0.608	0.136	2.792	0.5163
0<VL^c^⩽1.29	3/10	1.186	0.322	4.372	0.7976
1.29<VL^c^⩽12.70	4/11	1.438	0.451	4.588	0.5397
12.70<VL^c^					
					
HPV 18/45	111/492	Ref.^b^	—	—	—
Negative	9/19	2.100	0.925	4.764	0.0760
0<VL^c^⩽0.65	7/21	1.477	0.613	3.561	0.3846
0.65<VL^c^⩽2.65	12/16	3.324	1.530	7.225	0.0024
2.65<VL^c^					

a Normalised viral load (number of HPV genomes per human cell equivalent).

b Reference group.

c Mean normalised viral load (number of HPV genomes per human cell equivalent) based on all HPV-positive smears per individual.
